# Type 1 diabetes risk factors, risk prediction and presymptomatic detection: Evidence and guidance for screening

**DOI:** 10.1111/dom.16354

**Published:** 2025-03-25

**Authors:** Ezio Bonifacio, Anette‐Gabriele Ziegler

**Affiliations:** ^1^ Center for Regenerative Therapies Dresden Technische Universität Dresden Dresden Germany; ^2^ Paul Langerhans Institute Dresden of the Helmholtz Munich at University Hospital Carl Gustav Carus and Faculty of Medicine TU Dresden Dresden Germany; ^3^ German Center for Environmental Health Institute of Diabetes Research, Helmholtz Munich Munich Germany; ^4^ Forschergruppe Diabetes e.V. at Helmholtz Munich German Research Center for Environmental Health Munich Germany; ^5^ Forschergruppe Diabetes, School of Medicine, Klinikum rechts der Isar Technical University Munich Munich Germany

**Keywords:** cohort study, cost‐effectiveness, population study, primary care, type 1 diabetes

## Abstract

Type 1 diabetes is recognized as a chronic disease with a presymptomatic phase that does not require insulin therapy and a clinical phase where insulin treatment becomes necessary. The presymptomatic phase is characterized by the presence of autoantibodies targeting pancreatic islet beta cell antigens (islet autoantibodies). This phase is further classified into three stages: Stage 1, defined by normoglycaemia; Stage 2, characterized by dysglycaemia; and Stage 3, marked by hyperglycaemia, which typically presents clinically and necessitates insulin therapy. The prospect of therapies to delay the onset of clinical disease and insulin treatment has been a driver of research into the presymptomatic phase since the discovery of islet autoantibodies. With the recent approval of teplizumab as a therapy to delay disease progression, attention has increasingly focused on diagnosing individuals with Stage 1 and Stage 2 type 1 diabetes. However, diagnosing an asymptomatic condition that affects fewer than 1 in 200 individuals poses significant challenges. As we enter this new era of diagnosis, it is crucial to refine diagnostic approaches to ensure accuracy and effectiveness. This review summarizes current evidence and guidance while emphasizing the need for continued research alongside broader application of screening.

**Plain Language Summary:**

Type 1 diabetes is an autoimmune disease that affects approximately 0.5% of individuals. In this publication, the authors provide a comprehensive overview of strategies for identifying individuals in the pre‐symptomatic, early stages of the disease. Early‐stage type 1 diabetes can be detected by the presence of autoantibodies against specific proteins in the blood, signaling an ongoing disease process before clinical symptoms appear. Genetic factors also contribute to the development of these autoantibodies and the disease itself. The paper explores how these markers are used for early identification, emphasizing optimal screening ages and the role of confirmation tests in preventing misdiagnosis. A key consideration in early diagnosis is that disease progression varies–some individuals develop clinical diabetes rapidly, while others may take many years. The authors discuss additional tests that can help predict how soon a diagnosed individual may require insulin treatment. Finally, the paper highlights ongoing challenges in optimizing screening for wider application and the complexities of integrating research‐based screening into routine clinical practice.

## 1 AUTOIMMUNITY AND EARLY‐STAGE TYPE 1 DIABETES

A hallmark of type 1 diabetes is the presence of islet autoantibodies.^1^ These autoantibodies target four main antigen groups: Insulin and proinsulin, the 65 kilodalton form of glutamic acid decarboxylase (GAD65), insulinoma antigen‐2 (IA‐2) and IA‐2β and zinc transporter 8 (ZnT8). While other islet autoantigens, such as tetraspanin 7,^2^ have been identified, autoantibodies against these are observed far less frequently than those targeting the four major antigen groups.^3^ Differences in beta cell localization, genetic associations and age‐related frequencies are evident among the islet autoantibodies, with key characteristics summarized in Table 1.

**TABLE 1:** Characteristics of islet autoantigens and autoantibodies found in early‐stage type 1 diabetes.

Autoantigen	Cell distribution	Islet cell localization	Autoantibody features			
			Age relationship	HLA class II association	Other disease associations	Specificity for progression to Stage 3
Insulin	Islet beta cells	Secretory granule	Peak incidence at age 1 year, declines with age, infrequent in adult‐onset type 1 diabetes	HLA DR4‐DQ8	None	High when in combination with other islet autoantibodies; Low when single, except in very young children
Glutamic acid decarboxylase (GAD_65_)	Hormone containing islet cells endocrine organs, central nervous system	Small synaptic‐like vesicles	Early peak incidence less pronounced than IAA, characteristic of adult‐onset type 1 diabetes	HLA DR3‐DQ2	Type 1 diabetes, neurological diseases, thyroid autoimmune disease, gut autoimmune disease	High when in combination with other islet autoantibodies; Low when single
Insulinoma Antigen‐2 (IA‐2)	Hormone containing islet cells endocrine organs, central nervous system	Secretory granules	Early peak incidence less pronounced than IAA, less frequent in adult onset type 1 diabetes	HLA DR4‐DQ8	None	High also when single
Zinc transporter 8 (ZnT8)	Islet beta cells	Secretory granules	No peak incidence, less frequent in adult‐onset type 1 diabetes	None within type 1 diabetes associated haplotypes	None	High when in combination with other islet autoantibodies; Low when single
Tetraspanin‐7	Hormone containing islet cells, endocrine organs, central nervous system, lung	Secretory granules	No peak incidence, less frequent in adult‐onset type 1 diabetes	HLA DR4‐DQ8	None	High when in combination with other islet autoantibodies; Unknown for single antibodies

Early studies reported a specific relationship between the presence of two or more islet autoantibodies and progression to clinical type 1 diabetes.^4^, ^5^ However, the landmark publication restulted by pooling data from over 13,000 individuals followed prospectively from infancy for up to 30 years across three major cohorts in Germany, Finland and the United States.^6^ These findings conclusively demonstrated that the presence of two or more islet autoantibodies in childhood was associated with an almost 100% likelihood of developing clinical type 1 diabetes by adulthood. This evidence established the foundation for defining the presymptomatic phases of type 1 diabetes into distinct stages,^7^ later incorporated into the International Classification of Diseases (ICD) codes.

1. Early‐stage presymptomatic type 1 diabetes (unspecified; ICD E10.A0): The presence of two or more of the four major islet autoantibodies, sub‐staged into:
2. Stage 1 (ICD E10.A1): The presence of two or more of the four major islet autoantibodies in individuals with normoglycaemia.
3. Stage 2 (ICD E10.A2): Defined by the presence of two or more islet autoantibodies along with dysglycaemia.
4. Stage 3: Marked by hyperglycaemia, often accompanied by clinical symptoms of type 1 diabetes and typically requiring insulin treatment. Stage 3 type 1 diabetes and the clinical diagnosis of type 1 diabetes, as defined by ADA criteria^8^ are not always identical. The classification Stage 3a has been proposed for hyperglycaemia that does not meet the ADA criteria for type 1 diabetes.^9^

A critical point is that early‐stage type 1 diabetes, particularly Stage 1, is diagnosed entirely based on laboratory tests. This makes the establishment of clear diagnostic criteria essential. Accurate criteria are vital to avoid overdiagnosis (false positives: individuals who do not develop clinical type 1 diabetes) and underdiagnosis (false negatives: individuals who develop clinical type 1 diabetes without an early‐stage diagnosis). In the strictest sense, true early‐stage type 1 diabetes should reliably progress to clinical type 1 diabetes, with diagnostic tests minimizing both false positives and false negatives.

## 2 RISK FACTORS FOR EARLY‐STAGE TYPE 1 DIABETES

### 2.1 Genetic risk

Type 1 diabetes is underpinned by polygenic susceptibility, as demonstrated by its increased prevalence among individuals with a genetic link to affected relatives.^10^ Decades of research have identified numerous genomic regions associated with increased susceptibility to type 1 diabetes, with the HLA class II region on chromosome 6 being the most prominent contributor.^11^ This region, particularly the HLA DRB1‐DQA1‐DQB1 genotypes, exhibits extensive polymorphism, with each genotype conferring a unique level of risk.^12^, ^13^ While some genotypes are highly susceptible to type 1 diabetes, others offer substantial protection, forming a spectrum of risk ranging from highly susceptible to highly protective.

Classic high‐risk genotypes are those containing an HLA DRB1*04‐DQA1*0301‐DQB1*0302 (DR4‐DQ8) or an HLA DRB1*03‐DQA1*0501‐DQB1*0201 (DR3‐DQ2) haplotype, with the risk reaching 5% in individuals with both these haplotypes.^12^, ^13^ Allelic variation in the gene encoding insulin (*INS*) also confers considerable risk for type 1 diabetes^14^ and modifies the risk associated with HLA class II genotypes.^15^ Both genetic regions predominantly affect the risk for developing early‐stage (multiple islet autoantibodies) type 1 diabetes.^15^, ^16^, ^17^, ^18^ Numerous other regions encoding genes expressed in immune cells and islet cells and involved in immune and anti‐viral responses confer additional risk.^19^, ^20^ Collectively, genetic susceptibility across all regions can be quantified using polygenic risk scores (PRS), which integrate information from multiple risk loci.^21^, ^22^, ^23^, ^24^ PRS not only enhance the ability to predict and diagnose type 1 diabetes, but may also prove valuable for screening early‐stage type 1 diabetes, aiding early identification and intervention.^25^, ^26^, ^27^

### 2.2 Modifiers of genetic risk

The background genetic risk for type 1 diabetes varies substantially across populations, influenced by geographic location (space) and ancestry. However, absent major disruptions such as widespread lethal infections, mass emigration or immigration, the genetic architecture of a population or ancestral group remains relatively stable over decades (time). In contrast, the incidence of type 1 diabetes has increased significantly over recent decades, indicating the influence of non‐genetic factors.^28^ Although data on early‐stage type 1 diabetes are limited, it is reasonable to infer similar trends. The identification of non‐genetic modifiers of risk has been less successful than the elucidation of the genetic architecture of type 1 diabetes. Nevertheless, clear and strong modifiers of genetic risk have been identified, and some of these are relevant to screening for early‐stage type 1 diabetes.

Age is likely the most significant modifier of genetic risk. The incidence of clinical type 1 diabetes varies by age, peaking around adolescence. More striking, however, is the age‐related onset of autoimmunity that defines early‐stage type 1 diabetes. Autoimmunity is rare in the first 6 months of life but peaks dramatically between 1 and 2 years of age, followed by a decline to a steady, lower incidence throughout childhood and into the teenage years.^29^, ^30^, ^31^ This age‐related pattern is largely determined by the development of insulin autoantibodies and is strongly associated with HLA genotypes, particularly the presence of HLA DR4‐DQ8.^29^, ^30^, ^31^, ^32^ However, the overall risk of developing islet autoantibodies declines exponentially between 6 months and 6 years of age for all HLA risk genotypes.^33^ Furthermore, the genetic risk associated with *INS* genotype and PRS also diminishes with age, making age a critical factor in modifying genetic susceptibility to early‐stage type 1 diabetes. This has important implications for determining the optimal timing of screening. Additionally, age interacts with the number and type of islet autoantibodies present in the presymptomatic phase of type 1 diabetes. Adults who progress to clinical type 1 diabetes often exhibit fewer islet autoantibodies, predominantly GAD antibodies, compared to children.^34^ Consequently, screening and diagnostic strategies may need to differ between adults and children.

Sex also influences genetic risk, primarily by modifying age‐related risk. Boys have a higher risk of developing early‐stage type 1 diabetes in the first few years of life, while girls with early‐stage type 1 diabetes progress faster to clinical disease.^6^, ^29^, ^35^ Although this finding may not directly impact screening strategies, it offers valuable insights into understanding disease progression and potential strategies for delaying its onset.

An intriguing and consistent observation is that children who are born to a mother with type 1 diabetes have approximately half the risk of developing early‐stage or clinical type 1 diabetes compared to children with fathers or siblings with type 1 diabetes but a non‐diabetic mother.^16^, ^36^, ^37^, ^38^ This relative protection is most pronounced during the early peak of seroconversion incidence. Since genetic susceptibility is similar in the offspring of mothers and fathers with type 1 diabetes, the protective effect likely arises from exposure to the maternal diabetic environment during pregnancy. Potential mechanisms include accelerated pancreatic islet development, enhanced immune tolerance to islet autoantigens or epigenetic modifications affecting type 1 diabetes susceptibility. This area remains an important avenue for further research.

Finally, infection can modify the risk of early‐stage type 1 diabetes. Certain viral infections such as Coxsackie B virus^39^, ^40^, ^41^ and, more recently, SARS‐CoV‐2^42^ have been reported to be associated with an increased incidence of islet autoantibodies in genetically susceptible young children. Infection rates may, therefore, influence the prevalence of early‐stage type 1 diabetes in specific age groups and populations. Although no causal relationship between viral infections and islet autoimmunity has been established, potential mechanisms behind the associations include direct infection of islet cells, metabolic or inflammatory stress of beta cells or the immune system, or molecular mimicry.

## 3 PRACTICAL CONSIDERATIONS FOR SCREENING

### 3.1 The importance of *a priori* risk—The case for multiple tests

**FIGURE 1:**
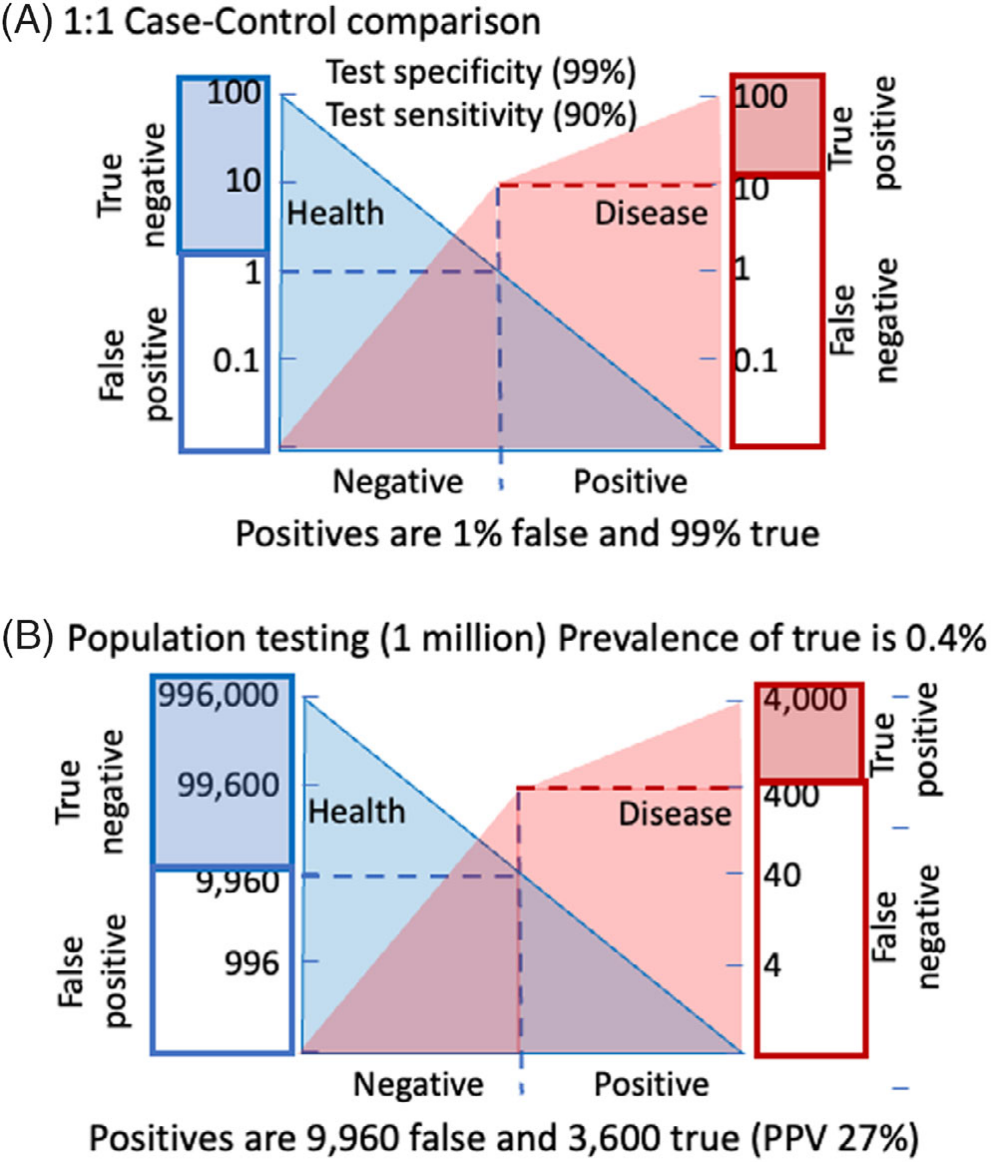
Effect of *a priori* probability on screening. The example shows the application of an islet autoantibody screening test that has a threshold for positivity set to the 99th centile of healthy controls (99% specificity). The threshold identifies 90% of the disease group (90% sensitivity). In a case‐control setting often used to evaluate the performance of a test (A), false positives are infrequent. Case B shows the performance of the same test in general population screening, where the *a priori* disease prevalence is 0.4% (4000 from 1 million tested). With 99% specificity in health and 90% sensitivity in disease, the test is expected to identify 3600 type 1 diabetes cases (90% of the total cases) plus another 9960 who will not develop type 1 diabetes, with a positive predictive value (PPV) of 27%.

Most medical diagnoses rely on multiple clinical or biomarker values to ensure certainty of disease. The mathematics are simple and based on Bayes' theorem,^43^ which, for our purpose, infers that the likelihood that a positive test accurately indicates disease increases when the test is applied in populations with high disease prevalence. For instance, a test for diagnosing disease in individuals with classic symptoms typically yields high diagnostic certainty. Conversely, applying the same test in a low‐prevalence population, such as screening for a disease with a prevalence of 3 in 1000, results in more false positives than true positives (Figure 1). The trade‐off between certainty (positive predictive value) and sensitivity (the proportion of true cases detected) often defines the diagnostic strategy. For severe, highly contagious diseases requiring immediate action, high sensitivity is prioritized, even at the cost of certainty. Conversely, in conditions where treatment can be delayed or has significant side effects, high diagnostic certainty is preferred, even at the expense of sensitivity. The first would require a test that identifies close to 100% of cases quickly and the second can perform multiple tests consecutively to reach a diagnosis. Diagnosing early‐stage type 1 diabetes falls into the high‐certainty category and is best achieved through multiple sequential tests.

### 3.2 Decision trees and the 2 × 2 × 2 principle to diagnose early‐stage type 1 diabetes

**FIGURE 2:**
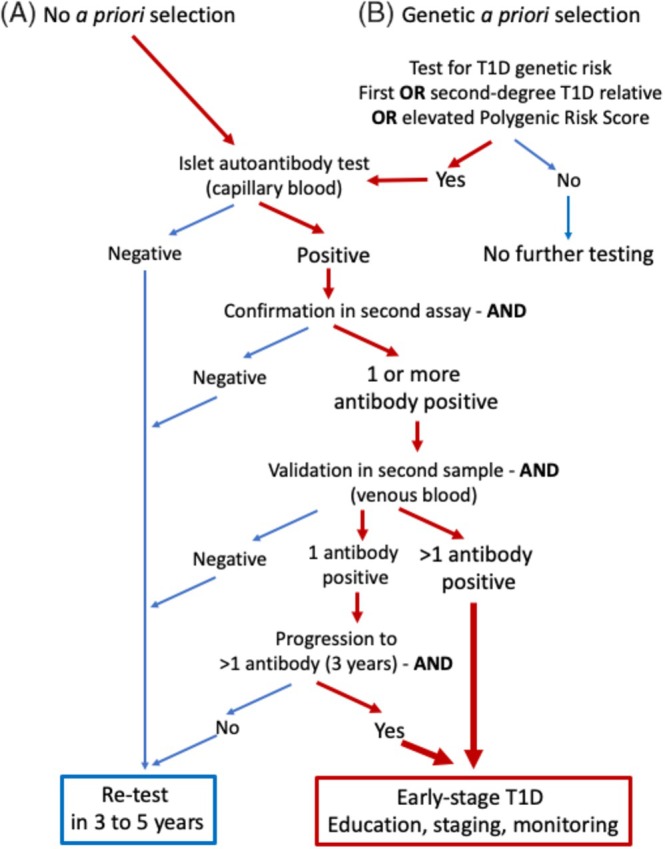
Early‐stage type 1 diabetes screening strategy decision tree options. Option A starts with islet autoantibody testing in all children with a series of AND commands leading to second assays and samples depending upon the screening test and confirmation test results. Option B includes an *a priori* selection based on genetic risk with OR commands that identify a subset of the population to be tested for islet autoantibodies following the AND command decision tree. The efficiency of screening, and in particular for option B, is dependent upon the recall rates achieved for autoantibody testing.

The application of sequential testing in screening resembles a decision tree with a series of AND and OR commands. Certain factors, such as family history of type 1 diabetes or age, can be considered early in the decision tree without requiring a sample or test. The sequence of tests is usually determined by factors such as cost, ease of application and test sensitivity. Highly sensitive tests are often used sequentially with AND commands, while less sensitive tests can be combined using OR commands to improve overall sensitivity. For example, ‘first‐degree relative with type 1 diabetes’ alone has low sensitivity but combining it with ‘PRS >90th percentile’ in an OR command yields high sensitivity for detecting early‐stage type 1 diabetes. Decision trees should be simple and practical. Two examples of childhood screening strategies are discussed (Figure 2).

The first approach begins with autoantibody testing. Timing is critical: screening too late misses cases of clinical type 1 diabetes that manifest early, while screening too early fails to capture those who seroconvert later. Current estimates^33^, ^44^, ^45^ suggest:

1. Screening at age 3 years identifies ~35% of cases progressing to clinical type 1 diabetes by age 18.
2. Screening at ages 2 and 6 years captures ~65% of cases.
3. Screening at ages 2, 6 and 10 years identifies approximately 80% of cases.

The choice of the autoantibody test is also important. For general population screening, the test must be cost‐effective, labour‐efficient and sensitive. Since 1985, the islet autoantibody standardization programme has provided a framework for assessing test performance.^46^ There are three assay methodologies currently in use for screening.

The EDENT1FI consortium in Europe follows the Fr1da experience^47^ and uses the 3Screen ELISA^48^, ^49^ as its first‐line screening test.^50^ It is the most validated for general population screening, yields high sensitivity for identifying those who should be further tested for early‐stage type 1 diabetes and is currently the least expensive alternative.^51^ It does not distinguish which of the three antibodies is positive, and it does not include insulin autoantibodies. However, it is an excellent screen to exclude over 98% of samples from further testing. The ASK study based in Colorado utilizes the ECL assay,^52^, ^53^ which has been an in‐house assay, but which is being worked up as a commercial assay by the company that specializes in these assays. The assay has performed well in workshops and in the hands of the Colorado investigators. It measures all four islet autoantibodies and distinguishes which of the antibodies are positive. The cost is substantially higher than the 3Screen ELISA, but it requires less blood volume. The third and most recently developed assay in use is the ADAP assay.^54^, ^55^ Like the ECL assay, it measures all four islet autoantibodies, distinguishes which are positive and requires a low sample volume. It is unknown how consistently it performs in multiple laboratories, and it is the most expensive of the three assays. Both the ECL and the ADAP assays can incorporate the measurement of additional autoantibodies and can also combine screening for islet autoantibodies with screening for autoantibodies associated with celiac disease and/or thyroid autoimmune diseases, approaches used by some investigators.^52^, ^53^, ^56^, ^57^ Sustainability with respect to the provision of reagents to satisfy the needs of general population screening is another aspect to consider when selecting assays.

Samples that are negative in the screen are no longer followed and can be excluded from the early‐stage type 1 diabetes diagnosis. Samples that are positive should be tested in a second different assay. For example, those that are 3Screen ELISA positive should be tested with one of the other assays or with other established and sensitive assays for each of the 4 islet autoantibodies. Similarly, samples positive in an ECL or ADAP assay should be re‐tested in assays with a different format. This does not require an additional sample. The need for confirmation with a second different assay has been questioned.^58^ However, our opinion is that at this still relatively early point, it remains an important step.^59^ It is not only good practice, but because the frequency of early‐stage type 1 diabetes is much less than 1%, tests are likely to detect false positives due to ‘non‐specific’ signals and these signals are likely to be assay specific.^60^ Children who are positive for two or more islet antibodies in their initial screening sample require confirmation with a second sample tested for all four autoantibodies. This step mitigates errors related to sample handling. Following the 2 × 2 × 2 rule—requiring at least two positive antibodies confirmed by two different tests on one occasion and subsequently in a second occasion—provides a robust framework for diagnosis. Using this strategy we have diagnosed early‐stage type 1 diabetes in 569 (0.28%) of 203 354 screened children aged 2–11 years in the Bavarian state of Germany. To enhance diagnostic accuracy further, incorporating genetic tests—such as PRS or family history of type 1 diabetes—at the confirmation stage may be considered. Combining genetic data, age and the number and type of islet autoantibodies enables a ‘certainty grade’ for diagnosis to be developed.

A second approach involves genetic preselection to reduce the total number of tests required for multi‐age screening.^61^ The strategy, if incorporated into newborn screening, will also allow detection of children diagnosed with type 1 diabetes in the first 2 years of life.^26^ However, this method is effective only if the genetic pre‐screening selects a substantial proportion of the population for follow‐up autoantibody testing and achieves a high recall rate. One reported attempt had a recall rate of less than 10%^62^ and the strategy requires optimization and further evaluation before it can be recommended. Such a strategy could use the OR command to select genetically at‐risk children based on family history, HLA or PRS who would proceed to islet autoantibody testing. The cost‐effectiveness of the genetic‐first versus antibody‐first approaches remains undetermined, particularly when accounting for counselling requirements for genetic screening results. A hybrid strategy—combining genetic and autoantibody testing with follow‐up testing at older ages for genetically at‐risk but autoantibody‐negative children—may also be a viable option.^26^

## 4 STAGING AND PROGRESSION TO CLINICAL DISEASE

The diagnosis of early‐stage type 1 diabetes is a step that identifies individuals who are likely to develop clinical type 1 diabetes later in life. Diagnosis activates and requires proper clinical care. A key aspect of this care, particularly in the context of potential therapies to delay progression, is the accurate diagnosis of Stage 1, which is associated with less than 20% 2‐year progression to clinical diabetes or Stage 2, which is associated with a 50% 2‐year progression rate. The defining distinction between these stages is glycaemic status: normoglycaemia in Stage 1 and dysglycaemia in Stage 2.

Over the past decade, the definition of dysglycaemia in early‐stage type 1 diabetes has evolved. Initially, it relied solely on impaired oral glucose tolerance test (OGTT) values and subsequent confirmation.^7^ Later, it incorporated impaired HbA1c values and aligned the definition of impaired fasting glucose with those used in pre‐type 2 diabetes.^8^ A comparison of diagnostic criteria has revealed significant differences in the rates of progression to Stage 3 type 1 diabetes depending on the number and type of impaired values.^63^ While this variability could raise concerns, it also presents an opportunity to improve risk stratification (Table 2). Indeed, risk within Stage 2 type 1 diabetes can be stratified on the basis of the number and persistence of glycaemia abnormalities as well as the combination of abnormalities by blood glucose and HbA1c.^63^ Nevertheless, the OGTT is influenced by several factors—some controllable and others not—and there is sub‐optimal compliance in some studies.^64^ Alternative markers such as c‐peptide values during an OGTT, proinsulin‐c‐peptide ratios and values derived from continuous glucose monitoring (CGM) devices have been investigated.^65^, ^66^, ^67^, ^68^ Although thresholds for dysglycaemia from these measurements have been proposed, this is still in its infancy, with substantial variability within and between studies, particularly for CGM.^69^, ^70^, ^71^, ^72^ Nevertheless, we expect that once standardized, CGM will become a useful component for staging and stratification of risk in early‐stage type 1 diabetes.

**TABLE 2:** Tests and criteria used to stratify the risk of progression to Stage 3 or clinical type 1 diabetes in individuals with early‐stage type 1 diabetes.

Applied tests	Criteria	2‐year progression	Reference
OGTT and HbA1c	Dysglycaemia^a^ by OGTT AND HbA1c	80%	58
	Two or more dysglycaemic values	67%	
	Single dysglycaemic value	27%	
	Dysglycaemia in consecutive timepoints	63%	
IA‐2A, OGTT and HbA1c	Progression likelihood score (PLS) >4.0 in Stage 1 T1D	50%	71
	PLS ≥ 0.5 to 4.0 in Stage 1 T1D	10%	
	PLS < 0.5	0%	
OGTT (glucose + c‐peptide)	Index60 > 1.0	40%	107
BMI & OGTT (glucose & c‐peptide)	Diabetes Prevention Trial Risk Score (DPTRS) >7	40%	66
HbA1c	≥5.7% or >10% increase	45%	58
IA‐2A	IA‐2A positive	18%	Frida study (unpublished)

^a^ Fasting plasma glucose 5·6–6·9 mmol/L (100–125 mg/dL), or 2‐h plasma glucose 7·8–11·0 mmol/L (140–199 mg/dL), or HbA1c 5·7–6·4%, or ≥10% increase in two consecutive HbA1c values.

### 4.1 IA‐2 autoantibodies and progression likelihood scores

Certain autoantibodies, particularly IA‐2 autoantibodies (IA‐2A), play a pivotal role in staging and predicting disease progression. The presence of IA‐2A—either alone or in combination with other islet autoantibodies—is consistently associated with a higher rate of progression to Stage 3 type 1 diabetes.^6^, ^73^, ^74^, ^75^, ^76^ For example, in the Fr1da study, the progression rate to clinical type 1 diabetes for individuals with early‐stage type 1 diabetes who are IA‐2A positive is 18% by 2 years. IA‐2A also stratifies risk effectively in both Stage 1 and Stage 2. Given its relative ease of measurement and the challenges associated with OGTT, IA‐2A can be a valuable tool for stratification. This is particularly relevant for families who may be averse to OGTT testing, where the use of HbA1c can be complemented by IA‐2A.^77^

The TrialNet group has proposed several scores to identify individuals at higher risk of progressing rapidly to clinical diabetes.^65^, ^78^ Many of these scores include additional C‐peptide measurements during OGTT, which provide significant value in stratifying Stage 2 type 1 diabetes. However, their utility in Stage 1 type 1 diabetes remains limited.

In our analysis, we examined parameters associated with faster progression in children with Stage 1 type 1 diabetes.^77^ From this, we developed a Progression Likelihood Score based on three markers:

1. IA‐2A autoantibodies
2. HbA1c levels
3. 90‐min glucose value from the OGTT

This score demonstrated impressive utility:

1. It identified a subset of Stage 1 individuals with progression rates similar to those with definitive Stage 2 type 1 diabetes.
2. It distinguished approximately 30% of children with Stage 1 diabetes who remained progression free for 2 years.

The identification of substages in early‐stage type 1 diabetes paves the way for more refined clinical tools. Decision trees, informed by stratification criteria such as the Progression Likelihood Score or decision trees based on recursive partitioning algorithms,^79^ could help clinicians tailor interventions. These decision trees could be further enhanced by algorithms or computer‐assisted programmes that assign individualized progression rates.^80^, ^81^ Together, these approaches represent a significant step forward in the early detection and management of type 1 diabetes, guiding clinical care by providing a more tailored decision‐making framework and offering new opportunities to delay progression and improve patient outcomes.

## 5 LIMITATIONS OF PREDICTION IN EARLY‐STAGE TYPE 1 DIABETES

### 5.1 Group versus individual risk

A common oversight when assessing risk is that the risk attributed to a group of individuals who meet certain criteria represents an **average risk**, not a precise prediction for any single individual. In most cases, we cannot pinpoint exactly when an individual within that group will progress to clinical diabetes. Therefore, caution is necessary when proposing the use of multiple variables to define a precise risk, as this risk should be understood as an estimate, not an exact forecast. It is important to avoid the misconception that, with enough information, we can precisely predict when someone will develop clinical diabetes.

Additionally, it is important to recognize that individuals within the same group may have different baseline parameters. If tests were sufficiently precise, it might be possible to more accurately predict an individual's progression by tracking changes in their baseline measures. This approach is partially used in defining Stage 2 diabetes, where progression is often indicated by an increase in HbA1c.^82^, ^83^

### 5.2 Research versus real‐world settings

The research setting strives to generate data that are precise and accurate. This includes central measurements using standard operating procedures. However, real‐world conditions are not as controlled as those in research or clinical trial settings. When screening and monitoring are conducted in public healthcare environments, there is an increased likelihood of variability in test results due to the diversity of assay providers and the larger number of centres that perform the testing. There are two undesired consequences of imprecise tests in this context. The first is misdiagnosis and the second is an inability to identify true change in an individual. Consequently, while certain findings may have value in a research environment, their applicability in real‐world clinical practice may be limited. Furthermore, it may not be practical to introduce complex algorithms for diagnosis and care into clinical practice. It will be crucial, therefore, to assess the effectiveness of our current approaches in practical settings and adapt accordingly.

### 5.3 The future may not mirror the past

Prediction models often assume that the future will follow the patterns of the past. However, the incidence of type 1 diabetes has varied considerably across the globe over the last 50 years.^28^ As such, what we have learned from studies conducted 20 or 30 years ago may not be entirely applicable to the present or to type 1 diabetes diagnoses in the coming decades.

Changes in the environment, such as the emergence of new viruses (e.g., the COVID‐19 pandemic), shifts in pollution levels and climate changes, all interact with genetic susceptibility and may influence the incidence and progression of type 1 diabetes. Moreover, the weight of specific susceptibility genes and the accuracy of PRS could change over time as environmental factors evolve.^84^ Therefore, prediction models should be regularly updated, and continuous research monitoring should remain an integral part of future clinical guidelines. By doing so, we can ensure that our predictive tools remain relevant and accurate in light of ongoing global and environmental changes.

### 5.4 Screening in adults

Much of the current understanding of early‐stage type 1 diabetes screening has focused on children and adolescents. However, around half of clinical type 1 diabetes diagnoses occur in adulthood.^34^ Some of these adults may have already developed multiple islet autoantibodies in childhood or adolescence, but it is likely that many would be missed (islet autoantibody negative) if they had been screened during childhood.

Screening in adulthood has been performed in reasonable numbers in relatives of patients,^85^ women with gestational diabetes,^86^ and in the general population.^87^ Screening has also been performed in adults with associated diseases such as thyroid autoimmune disease.^88^ The messages that emerge are that we have fewer islet autoantibodies to rely on to identify early‐stage type 1 diabetes in adults and progression to clinical type 1 diabetes appears to be slower than in children with early‐stage type 1 diabetes. In adults, insulin autoantibodies (a key marker in childhood) are infrequently detected, and the autoantibody profile often consists primarily of single GAD autoantibodies.^34^ These antibodies are less specific for type 1 diabetes and are found in other autoimmune diseases.^89^, ^90^ In children with pre‐existing genetic risk, the presence of single GAD autoantibodies is associated with a 20% progression to clinical disease over a 15‐year period.^6^ Given this, identifying true early‐stage type 1 diabetes in adults requires multiple layers of testing, including immune, genetic and metabolic markers, and further research is needed to refine these strategies.

### 5.5 Single islet autoantibodies

There is ongoing debate about whether the presence of a single islet autoantibody warrants monitoring for early‐stage type 1 diabetes. The controversy stems from balancing the risk of missing individuals who may progress to clinical disease with the potential for over‐notifying individuals who will never develop type 1 diabetes.

Progression rates for individuals with single islet autoantibodies vary depending on the specific islet autoantibody present, the individual's age and genetic risk.^25^, ^91^, ^92^ It is well established that the highest progression rates are seen in those with single IA‐2 autoantibodies, high‐affinity antibodies, higher antibody titres, younger age and an elevated genetic risk for type 1 diabetes.^6^, ^75^, ^91^, ^92^, ^93^ In children with elevated genetic risk for islet autoantibodies, progression to early‐stage type 1 diabetes (manifested by multiple islet autoantibodies) usually occurs within 2–3 years from seroconversion to single islet autoantibodies.^94^, ^95^ For children with single islet autoantibodies, further genetic testing may be considered. For those with single islet autoantibodies, elevated genetic risk and normoglycaemia, monitoring for progression to multiple islet autoantibodies should continue for 3 years and could be achieved using capillary blood samples. If no progression occurs and the individual remains positive for a single islet autoantibody, further monitoring may be stopped.

## 6 COUNSELLING, EDUCATION AND MONITORING

The diagnosis of early‐stage type 1 diabetes must be accompanied by appropriate care. Several consensus groups have proposed guidelines, ranging from minimal care to more intensive research protocols.^9^, ^96^, ^97^ A practical framework for care is one that considers the likelihood of imminent progression to clinical type 1 diabetes while minimizing the intensity of testing for individuals.

Counselling and education are often underappreciated aspects of clinical care. One of the challenges we face, particularly with genetic pre‐screening, is the substantial number of individuals who require counselling after receiving information about their elevated risk for type 1 diabetes. While we acknowledge the potential negative psychosocial effects of informing families that their child may have a disease with no current symptoms^47^, ^98^, ^99^, ^100^—one that may not manifest for many years—there are also clear benefits to early‐stage diagnosis.^101^ If islet autoantibody testing is done properly, the majority of those diagnosed (approximately 0.3%–0.5% of the population) will eventually develop clinical type 1 diabetes. This is not the case for genetic pre‐screening, where 10%–20% of screened individuals may be selected for follow‐up testing and informed about a risk for type 1 diabetes that is 1.5%–2.5%, leading to more widespread counselling.

## 7 THE FUTURE AND WHAT IS STILL NEEDED

Islet autoantibody screening has now been successfully rolled out in several countries, including Italy, where a law allows for reimbursement of screening costs.^47^, ^56^, ^57^, ^102^, ^103^, ^104^ However, there remain several valid criticisms and concerns. Our stance has always been that screening will inevitably become more widespread, and we must be prepared to implement it with care and least harm for those being screened. As treatment for early‐stage type 1 diabetes is approved in additional countries, screening will likely become more routine. Without clear guidelines and a well‐structured and informed screening approach, there is a risk of misdiagnosis. Fortunately, there have been considerable efforts in studying genetically at‐risk individuals, as well as the general population, to guide the screening process so that its introduction into regular care can proceed. Nevertheless, many important questions remain unanswered, and several areas still need improvement. As such, it is crucial that study protocols continue to run parallel to screening activities. Areas for further investigation include the following:

1. Assess the harm and changes in behaviour associated with screening and identifying early‐stage type 1 diabetes.
2. Accurately determine the cost and cost benefit of screening for delaying clinical type 1 diabetes onset or reducing complications and hospitalization at presentation. This includes efforts to reduce costs and increase the ease of screening assays and procedures such as introducing less invasive tests without compromising accuracy.
3. How to most effectively perform screening to identify those who will develop clinical diabetes in adulthood, with consideration of whether to notify, counsel and monitor those with single islet autoantibodies.
4. Adapt screening to globally diverse populations and settings. Factors such as ethnic differences, genetic variability and environmental influences can impact the effectiveness of screening strategies. It is crucial to tailor screening protocols to different regions and populations to ensure the highest accuracy and relevance.
5. Guidelines, communication and infrastructure required for a safe and efficient transition into real‐world testing.

Finally, screening for early‐stage type 1 diabetes has mostly been conducted in research study environments. In regions like Bavaria, Germany, about 25% of the childhood population has been screened, but in other areas, screening coverage reaches less than 1% of the population. Expanding this to a larger, more comprehensive scale will introduce challenges that require:

1. New training programmes for healthcare providers.
2. More staff to manage screening efforts.
3. Significant investment in clinical care infrastructure.

If we are committed to the value of early‐stage type 1 diabetes screening, we must not only increase the number of individuals screened, but also ensure that we have the necessary resources and infrastructure to care for those identified.^105^ This will be a complex, large‐scale effort that requires coordination between healthcare systems, research institutions and policymakers.

## 8 CONCLUSION

Significant progress has been made in understanding early‐stage type 1 diabetes and establishing screening protocols, which allow screening for early‐stage type 1 diabetes to be introduced into regular health care. Alongside this, much work remains to ensure that screening is applied effectively and equitably. It will be essential to continue research into the psychological, economic and global aspects of screening, as well as to address the logistical challenges associated with its widespread implementation. If we aim to maximize the benefits of early detection and delay clinical onset,^106^ coordinated efforts and substantial investment are necessary to ensure the success and sustainability of these screening programmes.

## FUNDING INFORMATION

This work was supported by the German Federal Ministry of Education and Research (FZK 01KX1818), the German Federal Ministry of Education and Research as part of the German Center for Diabetes Research (DZD e.V.) and the German Center for Child and Adolescent Health (DZKJ, #01GL2406C), the Bavarian Ministry of Economic Affairs, Energy, and Technology (grant: Prevention of Autoimmune Diabetes–Digital Lab), the Novo Nordisk Foundation NNF22SA0081044, Breakthrough T1D (grant no 1‐SRA‐2014‐310‐M‐R) and the Deutscher Diabetiker Bund e.V. EB and AGZ are part of EDENT1FI, which is supported by the Innovative Health Initiative Joint Undertaking (IHI JU) under grant agreement No 101132379.

## CONFLICT OF INTEREST STATEMENT

EB and A‐GZ have received speaker's honoraria from Sanofi.

## PEER REVIEW

The peer review history for this article is available at https://www.webofscience.com/api/gateway/wos/peer‐review/10.1111/dom.16354.

## Data Availability

This is a review article with suggested guidelines and does not present original data.
